# Early Rectal Cancer: Diagnostic Challenges and the Role of Endoscopic Intermuscular Dissection Within the Therapeutic Algorithm

**DOI:** 10.3390/diagnostics16121936

**Published:** 2026-06-22

**Authors:** Rossella Maresca, Giulio Calabrese, Franziska Deutschbein, Valentina Blasi, Tommaso Schepis, Daniele Salvi, Silvia Pecere, Paola Cesaro, Cristiano Spada, Sandro Sferrazza, Federico Barbaro

**Affiliations:** 1 Digestive Endoscopy Unit, Fondazione Policlinico Universitario Agostino Gemelli IRCCS, 00168 Rome, Italy; rossella.maresca12@gmail.com (R.M.); federico.barbaro@policlinicogemelli.it (F.B.); 2 Gastroenterology Unit, San Pio Hospital, 82100 Benevento, Italy; 3 Department of Clinical Medicine and Surgery, University of Naples Federico II, 80125 Naples, Italy; giuliocalabrese94@gmail.com; 4 Department of Gastroenterology and Endoscopy, Fondazione Poliambulanza Istituto Ospedaliero, 25124 Brescia, Italy; daniele.salvi@poliambulanza.it (D.S.);; 5 Gastroenterology and Endoscopy Unit, ARNAS Civico-Di Cristina-Benfratelli, 90127 Palermo, Italy

**Keywords:** early rectal cancer, endoscopic intermuscular dissection, locoregional staging, deep submucosal invasion, magnetic resonance imaging, endoscopic ultrasound, organ-preserving

## Abstract

Early rectal cancer represents a challenging setting in which accurate locoregional staging is essential to guide appropriate treatment. Current diagnostic strategies primarily include magnetic resonance imaging (MRI) and endoscopic ultrasound (EUS). However, both modalities show significant limitations in early-stage disease, particularly in T staging. This diagnostic gap impacts therapeutic decision-making, particularly in patients with lesions suggestive of deep submucosal invasion. In these cases, endoscopic submucosal dissection (ESD) may be insufficient to achieve adequate vertical negative margins, whereas radical surgery is associated with considerable morbidity and potential impairment of quality of life. In this gray zone, endoscopic intermuscular dissection (EID) has recently emerged as a novel therapeutic approach designed to overcome the limitations of standard endoscopic resection. By enabling dissection within the deeper intermuscular plane, it can achieve curative resections while preserving rectal wall integrity. This narrative review aims to explore the current diagnostic gaps in early rectal cancer and to define the potential role of EID within the current therapeutic algorithm.

## 1. Introduction

Colorectal cancer (CRC) is the third most common cancer and the second leading cause of cancer-related deaths worldwide. Rectal cancer accounts for approximately 40–50% of all CRC diagnoses [1,2] and shows distinct molecular, biological, and clinical features from colonic cancer, thereby necessitating different management approaches [3].

The widespread implementation of colorectal cancer screening programs has led to an increased detection of early stage rectal cancer, mostly without nodal involvement, thereby expanding the need for organ-preserving treatment strategies [4,5]. Treatment decision-making for rectal cancer is primarily guided by the TNM staging. Early-stage rectal cancer generally comprises stage 0 (TisN0M0) and stage I (T1–T2N0M0) disease and is characterized by the absence of regional lymph node involvement and distant metastases. Specifically, Tis lesions are confined to the mucosa, T1 tumors invade the submucosa and can be further subclassified according to the depth of invasion, whereas T2 tumors extend into the muscularis propria without breaching it [6]. While early stage disease can be effectively managed by organ-preserving approaches, including endoscopic techniques (i.e., endoscopic submucosal dissection—ESD), and minimally invasive transanal surgical procedures (i.e., transanal endoscopic microsurgery—TEM and transanal minimally invasive surgery—TAMIS), more advanced stages require radical surgery with transabdominal resection with total mesorectal excision (TME) [7]. Despite its durable oncological control, radical surgical resection is associated with significant mortality and morbidity and may negatively impact quality of life [8,9]. In fact, TME is associated with long-term functional impairment, including urinary, sexual, and bowel dysfunction, such as low anterior resection syndrome, and may require temporary or permanent stoma formation [10]. Therefore, accurate staging of early rectal cancer is essential to avoid overtreatment.

Careful endoscopic assessment plays a pivotal role in the diagnostic work-up of early rectal cancer, as advanced endoscopic imaging techniques, including high-definition endoscopy, chromoendoscopy, and narrow-band imaging (NBI), may help predict the depth of submucosal invasion and guide therapeutic decision-making [11]. According to the guidelines, locoregional staging is mainly based on two modalities: endoscopic ultrasound (EUS) and magnetic resonance imaging (MRI). MRI is the diagnostic gold standard for locally advanced rectal cancer. However, its accuracy in differentiating T1 from T2 cancer remains highly debated, and growing evidence suggests a growing role of EUS in improving specificity in early-stage cases [12]. Advanced endoscopic resection techniques, such as ESD, have been proposed as standard local treatments for early rectal cancer. Endoscopy may represent a valuable option with curative intention for the treatment of early disease in the absence of any high-risk factors for lymph node metastasis such as deep submucosal invasion (>1000 um), lymphovascular invasion, poorly differentiated adenocarcinoma, or high-grade tumor budding [13,14]. However, ESD may not reliably achieve curative outcomes in lesions with severe fibrosis or deep submucosal invasion. Furthermore, emerging evidence suggests that deep submucosal invasion (>1000 µm, sm2–3) alone is not an independent risk factor for lymph node metastasis, with an absolute risk of lymph node metastasis of only 2.6% [15]. Therefore, selected lesions with deep submucosal invasion (>1000 µm) could still be amenable to curative endoscopic treatment; however, conventional ESD may be technically challenging in these cases because of the limited dissection plane and the difficulty in achieving adequate vertical margins. Recognizing the need for a technique capable of overcoming the limitations of ESD while avoiding the life-altering impact of total TME in rectal lesions, Moons et al. first introduced endoscopic intermuscular dissection (EID) in 2018 [16] for the treatment of selected T1 rectal cancer with deep submucosal invasion. This new endoscopic approach showed that dissection between the circular and longitudinal layers of the muscularis propria could achieve R0 resection and may improve the likelihood of curative resection of even T1 rectal cancers with significant submucosal involvement, while maintaining rectal integrity. Nevertheless, EID is not yet included in standard guideline recommendations, and its role in the current diagnostic and therapeutic pathways remains unclear [17].

In this narrative review, we discuss the current diagnostic gaps in early rectal cancer, particularly in patients with suspected deep submucosal invasion, and explore the role of endoscopic intermuscular dissection in modern treatment strategies.

## 2. Materials and Methods

This article is a narrative review. We conducted a comprehensive structured search across PubMed, Scopus, and Medline, including only English-language articles published until March 2026. We used combinations of the following terms: “early rectal cancer,” “t1,” “rectal cancer,” “deep submucosal invasion,” “endoscopic submucosal dissection,” “locoregional staging,” “MRI,” “accuracy,” “endoscopic ultrasound,” “limits,” “advanced endoscopic techniques,” “organ-preserving strategy,” “endoscopic intermuscular dissection,” and “diagnostic performance.” Studies were included if they consisted of original research articles, including prospective or retrospective clinical series, descriptive case reports and comparative reviews, provided that the latter reported extractable clinical outcome data. Moreover, priority was given to large cohort studies, meta-analyses, randomized trials when available, and recent guidelines from major international societies. We also manually searched the reference lists of the selected studies and related reviews to detect any other relevant publications.

## 3. Diagnostic Challenges and Staging of Early Rectal Cancer

The best management for early rectal cancers (T1/T2 with no nodal involvement) is still under debate and is frequently discussed at multidisciplinary tumor board meetings. Notably, high-quality endoscopy has demonstrated good diagnostic performance for the optical diagnosis of T1 colorectal cancer [18,19,20]. Specifically, recent evidence has shown a moderate sensitivity for the optical diagnosis of T1 CRC with higher specificity for endoscopic unresectable lesions [21]. However, a substantial proportion of lesions initially considered as invasive cancers are later downgraded after histopathological evaluation, highlighting the limitations of optical diagnosis alone [21,22]. Although high-quality optical diagnosis plays a pivotal role in the assessment of these lesions, accurate locoregional staging remains challenging. Distinguishing superficial from deeply invasive tumors and predicting nodal involvement are essential to guide the choice of the correct therapeutic strategies. In this setting, MRI and EUS currently represent the main imaging modalities for local staging. However, both techniques still show important limitations in early rectal cancer, particularly in the assessment of T stage, with potential implications for treatment selection and the risk of both overtreatment and undertreatment.

### 3.1. Role of Magnetic Resonance Imaging (MRI)

MRI is regarded as the gold standard for rectal cancer staging as it provides a comprehensive evaluation of the mesorectum, lymph node status, and entire pelvis [7,17]. However, differentiation between T1 and T2 early rectal tumors remains the most difficult challenge and may occasionally result in overstaging [23], although diagnostic accuracy has been shown to improve when MRI is interpreted by experienced radiologists in specialized centers [23]. The performance of MRI for T staging in early disease remains suboptimal, with reported accuracy values often less than 40% [12,24], and it may be influenced by several factors. First, MRI provides inadequate visualization of the rectal submucosa, and a polypoid morphology of the lesion may limit diagnostic accuracy in T staging [25]. Furthermore, although MRI is widely regarded as less operator-dependent, compared to other radiological techniques, emerging evidence suggests that accurate T staging is still significantly associated with referral center expertise [12]. However, recent advances in MRI, including contrast-enhanced imaging and structured reporting, have improved its diagnostic performance. Contrast-enhanced MRI outperformed endoscopic ultrasound for Tis–T1 staging, achieving a high discriminatory power with an area under the curve (AUC) of 0.91 [26]. Moreover, including anatomical criteria such as the assessment of ≥1 mm preserved muscularis propria through structured reporting approaches achieved an accuracy of approximately 80%, even among non-expert radiologists [27].

As the presence of nodal metastases at preoperative staging represents an indication for neoadjuvant therapy, accurate lymph node assessment is of paramount importance. MRI showed only moderate accuracy in nodal assessment, with high specificity and suboptimal sensitivity [23,24,28]. A recent study reported limited MRI accuracy for nodal staging (high specificity >90%, but low sensitivity, ranging between 26 and 56%), leading to a risk of underdetection of lymph node metastases and potentially resulting in missed indications for neoadjuvant therapy and consequent undertreatment [29]. However, despite its limited sensitivity for nodal detection, MRI has been shown to outperform EUS in N staging, with pooled sensitivity and specificity of 83% and 90%, respectively [30].

Overall, the limited diagnostic accuracy of MRI may lead to a misclassification of disease status, ultimately impacting clinical decision-making.

### 3.2. Role of Endoscopic Ultrasound (EUS)

According to the latest guidelines, the use of EUS is generally reserved for patients unsuitable for MRI or cases where MRI findings are inconclusive or suboptimal [7,17,31]. Accurate staging is critical, as overstaging benign lesions may lead to overtreatment, whereas understaging early submucosal invasion may compromise oncological radicality.

Reported accuracy of EUS for T staging significantly varies across studies [32,33,34,35]. Compared with pathology reports, EUS has shown pooled sensitivity and specificity of 87.8% and 98.3%, respectively, for T1 staging [35]. A prospective study further showed higher diagnostic accuracy of EUS for T1 compared with T2 staging (82% compared to only 58%, respectively) [36]. These findings were supported by a meta-analysis showing that MRI was superior to EUS for T2 staging (*p* = 0.01), while EUS outperformed MRI for T1 staging [30]. Moreover, linear EUS has been shown to outperform radial EUS in the staging of deep submucosal invasive cancer, with significantly higher diagnostic accuracy (0.936 vs. 0.655, *p* = 0.003) and specificity (0.963 vs. 0.659, *p* = 0.003) [37]. Despite these promising results, the clinical reliability of EUS remains limited.

A recent retrospective study conducted in 2026 including 1000 consecutive EUSs performed before TEM, reported an overall diagnostic accuracy of EUS of 78.7%. When applying a threshold (≥pT1sm2), EUS achieved only moderate overall accuracy (approximately 65%), with good sensitivity (~76.6%) but limited specificity (~61.7%). The high negative predictive value (~89.9%) supports its role in excluding deep submucosal invasion. However, the low positive predictive value (~37.1%) reflects a significant tendency toward overstaging superficial lesions [38]. Ultimately, the timing of examination may also influence the performance of EUS, with a lower reported accuracy following prior endoscopic resection (44.3%) [38]. Overall, EUS appears to provide superior accuracy for T staging compared with MRI, particularly in early disease [30]. A recent nationwide 2026 multicenter cohort reported only moderate accuracy of EUS for T staging in T1 rectal cancer (59%). Although EUS outperformed MRI, both modalities demonstrated a significant tendency toward overstaging, particularly when used in combination [12].

Regarding N staging, EUS demonstrated moderate diagnostic accuracy in early rectal cancer. In the largest meta-analysis reported in literature, pooled sensitivity and specificity were 73.2% and 75.8%, respectively, while the area under the curve (AUC) of 0.79 indicated only moderate discriminatory ability [39]. Moreover, EUS can only detect mesorectal nodes in close proximity to the rectal wall and tumor site and may miss micrometastases [40]. Therefore, inaccurate staging may result in inappropriate treatment selection, including potential undertreatment. This concern is further supported by a multicenter study of 86 patients staged as T2N0, in which EUS excluded nodal metastasis with a negative predictive value (NPV) of 87.2%, with approximately 13% of cases of occult nodal metastases [41]. In this setting, operator experience represents another important factor influencing EUS performance. In fact, operator experience has been reported as the single most important predictor of EUS N staging, with experienced operators achieving significantly more accurate nodal staging (*p* = 0.01) and lower overstaging (*p* = 0.02) compared with non-experts, and showing an overall N0 staging accuracy of 63% [36].

Taken together, these findings highlight that neither EUS nor MRI alone provides consistently reliable staging in early rectal cancer. Therefore, combining EUS and MRI has emerged as a promising strategy to improve diagnostic accuracy and better guide treatment decision-making.

### 3.3. Potential Role of Combined EUS and MRI

Given the limitations associated with the isolated use of MRI and EUS, several studies have investigated whether a combined imaging approach could improve locoregional staging accuracy in rectal cancer, particularly in early-stage disease. In a large retrospective population-based study including patients with T1 colorectal cancer, MRI accuracy appeared lower than EUS for T staging (28.3% vs. 59%) [12]. However, combining modalities overstaged 67.1% of tumors for T staging. Concerning nodal staging accuracy, combined assessment showed high specificity (90.4%) but low sensitivity (13%), underlining the persistent difficulty in detecting occult nodal disease in early rectal cancer [12].

Overall, current evidence suggests that combining EUS and MRI may improve locoregional staging in early rectal cancer by increasing sensitivity for superficial tumor detection (see Table 1). However, this potential advantage may be accompanied by reduced specificity, with a consequent risk of overstaging that may lead to incorrect treatment selection. Further studies are therefore needed to better define the patient subgroups that may benefit most from combined imaging and to clarify its role in routine clinical practice.

## 4. Rationale and Technical Principles of Endoscopic Intermuscular Dissection

The concept of EID arises from the need to overcome the limitations of standard ESD in rectal lesions with severe fibrosis, a muscle-retracting sign (MRS), or suspected deep submucosal invasion. The technique was initially introduced as peranal endoscopic myectomy (PAEM) by Rahni et al. [42], who described a rectal lesion with severe fibrosis and an MRS treated with myotomy through the inner circular muscle, followed by dissection between the inner circular and outer longitudinal muscle layers. In a subsequent PAEM series by Toyonaga et al. [43], 10 rectal lesions with severe fibrosis and MRS were treated; en bloc resection with a negative vertical margin was achieved in 8/10 cases (80%), including mucosal lesions, shallow submucosal cancers, deep submucosal cancers, and one muscle-invasive lesion, with no significant post-procedural complications. The term EID was later adopted and clinically developed by Moons et al. [16], who applied this concept specifically to rectal cancers with optical suspicion of deep submucosal invasion. In their prospective cohort of 67 patients, EID achieved technical success in 64/67 cases (96%), R0 resection in 54/67 (81%), and curative resection in 30/67 (45%), with only minor adverse events reported in eight patients (12%). Importantly, among pT1 cancers with deep submucosal invasion, the R0 resection rate reached 90%, supporting the role of EID as a high-quality local excision and staging technique in carefully selected patients [16].

From a technical standpoint, EID is defined by the intentional shift of the dissection plane from the submucosa to the intermuscular space of the rectal wall. After mucosal incision and submucosal dissection to expose the muscularis propria, a selective myotomy of the inner circular muscle is performed, allowing access to the plane between the circular and longitudinal muscle layers. Dissection is then continued within this space, enabling en bloc removal of the lesion together with the full submucosal space and the underlying circular muscle, while preserving the outer longitudinal layer whenever possible. The pocket-creation method (PCM), already used in the early PAEM experience, may facilitate this process by providing stable access, intrinsic traction, and close exposure of the submucosal–muscular interface, helping to identify the MRS and redirect the dissection when the conventional submucosal plane is obliterated. Intermuscular tunnelling was subsequently proposed as a further refinement, using oral and anal mucosal incisions and anal-side tunnel entry to improve access, countertraction, and visualization of the muscular planes [44]. Although these technical adaptations may improve orientation and stability, EID remains technically demanding because the intermuscular plane is narrow and requires precise recognition of circular and longitudinal muscle fibers.

In selected cases with severe muscle retraction, suspected muscularis propria involvement, or inability to safely maintain the intermuscular plane, partial or intentional full-thickness resection may be required. This approach should be distinguished from EID, because full-thickness resection involves the entire rectal wall and may have different implications for defect closure, pelvic inflammation, and subsequent completion surgery. In this context, Argenziano et al. recently described knife-assisted full-thickness resection (kaFTRD) guided by the pocket-detection method for posterior deeply invasive rectal cancer [45]. This technique uses a submucosal pocket directed toward the suspected deeply invasive component to identify and isolate the MRS in real time. After circumferential isolation of this component, the muscularis propria is incised at least 3 mm from the MRS, and full-thickness resection is completed by dissecting the muscularis propria from the perirectal fat, followed by defect assessment and closure when required. In their initial series of four posterior-lateral rectal lesions, technical success, accuracy of detecting deep invasion, and en bloc resection were all 100%; no adverse events occurred, and after refinement of the technique to include a ≥3-mm muscularis propria margin, R0 resection was achieved in the three pT1bsm3 cases.

EID should be regarded as a distinct resection strategy rather than merely deeper extension of conventional ESD dissection. By converting the deep margin from a residual submucosal margin into a muscular margin, EID removes the deepest invasive front with a cuff of underlying circular muscle, while preserving the outer longitudinal layer whenever possible. This provides a specimen that is anatomically deeper than that obtained with ESD but less disruptive than full-thickness excision, offering a compromise between improved local radicality and preservation of rectal wall integrity. This intermediate position is central to its rationale: EID seeks to improve vertical margin clearance and pathological staging without completely disrupting the rectal wall. Nevertheless, whether this anatomical preservation translates into easier or safer completion surgery compared with full-thickness local excision remains uncertain and warrants specific evaluation in future studies.

**Figure 1:**
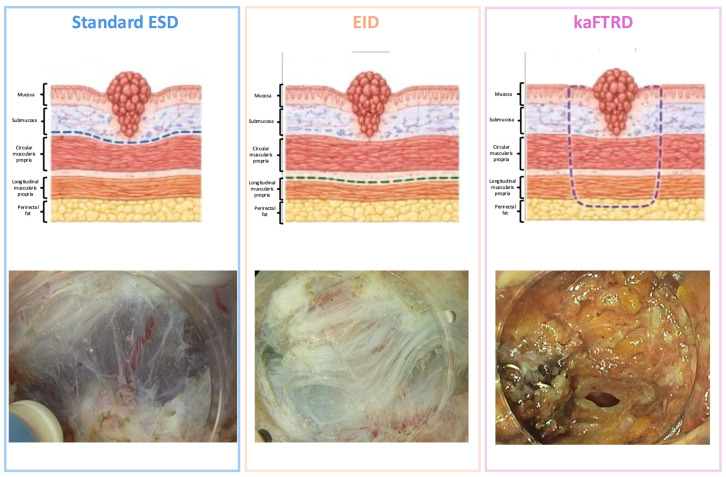
Schematic representation of the different dissection planes achieved by standard endoscopic submucosal dissection (ESD), endoscopic intermuscular dissection (EID), and knife-assisted endoscopic full-thickness resection (kaFTRD) in rectal lesions. In standard ESD, the dissection plane is confined to the submucosal layer above the muscularis propria, allowing en bloc resection of superficial lesions while preserving the muscular layer. EID extends the dissection deeper into the intermuscular space, between the circular and longitudinal layers of the muscularis propria, enabling resection of lesions with suspected deeper invasion while maintaining the integrity of the outer rectal wall. In contrast, kaFTRD achieves a transmural resection including the full thickness of the rectal wall up to the perirectal fat. Representative endoscopic views of the post-resection defects are shown in the lower panels.

As shown in Figure 1, ESD, EID, and kaFTRD differ substantially in the depth of wall resection, reflecting their distinct indications and potential oncological implications.

The principal oncological value of EID lies in pathology. By providing an en bloc specimen that includes mucosa, the full submucosal compartment, and the underlying muscle, EID improves assessment of the deepest invasive front and vertical margin, while preserving evaluation of standard risk factors such as lymphovascular invasion, tumor budding, differentiation, and lateral margin status. Its role is therefore not to replace oncological surgery or address mesorectal lymph-node disease, but to improve the quality of local excision and provide definitive histological risk stratification. Patients with positive margins, high-risk histological factors, suspected T2 disease, or suspicious lymph nodes remain candidates for completion treatment. From a technical and anatomical perspective, two issues remain particularly relevant when positioning EID within organ-preserving strategies: first, whether dissection at the level of the muscularis propria may influence the difficulty or safety of subsequent completion with surgery; second, whether the optimal anatomical indication should be limited mainly to the mid-to-distal rectum, where the muscularis propria is thicker and the risk of peritoneal perforation is lower than in the proximal rectum or rectosigmoid junction. These considerations define the framework within which current indications and clinical outcomes of EID should be interpreted.

Formal curricula and learning-curve studies for EID are currently lacking. Moreover, available evidence is largely derived from highly selected expert centers, making it difficult to define a reproducible case-volume threshold for competence. This limitation should be interpreted in the broader context of advanced ESD training, where substantial supervised exposure is required before achieving expert-level performance. In the series by Yamamoto et al. [46], trainee outcomes became comparable to those of experts only after more than 80 supervised ESD procedures. However, EID should be considered a step beyond conventional colorectal ESD, and competence cannot be assumed simply after reaching this threshold. Rather, EID should be acquired and performed in highly experienced referral settings by endoscopists who have already mastered colorectal ESD, including lesion assessment, dissection strategy, hemostasis, management of deep mural injury, and multidisciplinary decision-making in rectal cancer.

From a safety perspective, available data suggest that adverse events after EID are not negligible, although most reported events appear manageable in expert centers. The initial prospective EID cohort reported minor adverse events in 8/67 patients (12%), whereas the largest multicenter series reported adverse events in 35/188 procedures (18.6%), including four cases (2.1%) classified as AGREE grade IIIa [47]. Reported events included rectal stenosis requiring dilation, delayed bleeding, conservatively managed pneumorectum, and one grade V septic event in a highly fragile patient. These rates should be interpreted in light of the absence of validated EID-specific quality benchmarks. In a recent international ESD training survey [48], respondents who defined an acceptable adverse-event benchmark generally favored low rates, mostly well below 15%. Taken together, these data support a cautious implementation of EID, restricted to expert centers with structured audit, multidisciplinary support, and careful patient selection.

## 5. Current Indications and Clinical Outcomes of EID

Since its introduction, EID has emerged as an organ-preserving technique not only for T1 rectal cancers, but also for fibrotic or non-lifting lesions and residual or recurrent disease after chemoradiotherapy. Moreover, comparative evidence between EID and ESD remains limited to small retrospective series [44].

### 5.1. Selected T1b Rectal Cancers

In colorectal cancer, resection strategy is largely dictated by the depth of tumor invasion within the rectal wall [49]. In the AJCC TNM staging system (8th edition), T1 tumors are defined as those invading the submucosa without extension into the muscularis propria. Among these, the presence of high-risk histologic features, including deep submucosal invasion (defined as ≥1000 μm beyond the muscularis mucosae), lymphovascular invasion, poor differentiation, or high-grade tumor budding, generally supports consideration of radical surgery because of the higher risk of lymph node metastasis. However, growing evidence suggests that deep submucosal invasion, when present as the sole pathological risk factor, may have a limited role in the risk of lymph node metastasis [14].

This has defined a subgroup of patients in whom the optimal management remains uncertain. In this “gray zone,” organ-preserving strategies, including EID, are increasingly being explored.

Rectum-preserving approaches offer several advantages over TME, as they allow treatment of the primary tumor while maintaining anatomical integrity and function. However, current endoscopic techniques also have limitations. Residual malignancy has been reported in up to 19% of cases following non-curative ESD in surgical specimens [50], highlighting the risk of incomplete resection in lesions with deep submucosal invasion. In this context, EID has been proposed as a potential alternative to conventional endoscopic techniques [15]. By enabling dissection within the intermuscular plane, EID allows deeper resection compared with standard ESD and may improve vertical margin clearance, which is a critical limitation in lesions with deep submucosal invasion. In the largest multicenter study to date, including 188 patients with suspected deep submucosal invasive rectal cancer, EID achieved en bloc and R0 resection rates of 94.1% and 82.5%, respectively. At 3-year follow-up, no distant recurrence or cancer-specific mortality was observed, and locoregional recurrence rates were low (7–13%) in patients managed with surveillance, with all recurrences successfully salvaged, supporting the feasibility of an organ-preserving approach in selected cases [47]. Although these results are encouraging, the available follow-up remains relatively short and is insufficient to establish long-term oncological equivalence with radical surgery.

While no studies have directly compared EID with transanal endoscopic surgery (TES) techniques such as TEM or TAMIS, several technical considerations deserve attention. TEM and TAMIS allow full-thickness excision of rectal lesions and have demonstrated high en bloc and R0 resection rates, particularly for lesions with suspected deep submucosal invasion or significant fibrosis, showing favourable oncological outcomes [38,51]. As mentioned above, EID is performed entirely endoscopically and aims to achieve a deeper vertical resection margin through dissection in the intermuscular plane while preserving the outer longitudinal muscle layer. Theoretically, this approach may reduce procedural invasiveness and avoid some of the morbidity associated with full-thickness rectal wall excision and defect closure [51]. On the other hand, EID requires precise identification of the intermuscular plane and advanced endoscopic expertise, and its learning curve, reproducibility, and long-term oncological outcomes remain largely undefined. Evidence from systematic reviews comparing ESD and TEM suggests that endoscopic approaches may be associated with shorter hospital stays and lower procedural morbidity while maintaining acceptable oncological outcomes in selected rectal lesions, although direct extrapolation of these findings to EID should be made with caution [38,51]. Furthermore, the ongoing TRIASSIC [52] trial comparing TAMIS and ESD for large non-pedunculated rectal lesions highlights the continued clinical interest in defining the optimal balance between endoscopic and transanal surgical approaches. Therefore, the relative advantages and limitations of EID compared with TEM and TAMIS remain speculative, highlighting an important gap in the current evidence.

Overall, EID may represent a potential intermediate option between conventional endoscopic resection and radical surgery in carefully selected T1b rectal cancer, potentially bridging the gap between oncological radicality and organ preservation. However, longer-term surveillance and prospective comparative studies are needed to better define the oncological role of EID.

### 5.2. Fibrotic or Non-Lifting Benign Lesions

Fibrotic or non-lifting benign lesions arise following prior interventions, including piecemeal resection, biopsy sampling, surgical manipulation, or radiation therapy and represent a common source of technical difficulty in standard endoscopic resection [53]. ESD relies on the creation of a submucosal lifting plane through injection; however, in the presence of fibrosis, this plane is often absent or severely distorted making dissection technically challenging, with reduced visibility, impaired traction, and an increased risk of perforation.

One of most common endoscopic indicators of fibrosis is the non-lifting sign, first described by Uno, which reflects the absence of a suitable submucosal dissection plane [53,54]. Given the high prevalence of fibrosis, reported in approximately 36–65% of colorectal lesions undergoing ESD [55,56,57,58], accurate recognition is essential to guide the selection of the optimal resection strategy. These limitations have prompted the exploration of alternative techniques capable of overcoming the challenges posed by fibrosis. In this context, EID allows dissection beyond the fibrotic submucosa by accessing the intermuscular plane, enabling controlled en bloc resection even when a clear submucosal layer is not identifiable [16,59]. Clinical evidence supporting the use of EID in fibrotic or non-lifting benign lesions is increasing. In a series by Tribonias et al., including 23 EID procedures, 12 cases involved benign scarred lesions, all characterized by severe fibrosis. En bloc resection was achieved in all cases, with no intraoperative or delayed perforations and no delayed bleeding [60]. Additional reports have shown that EID can achieve complete resection with favorable safety outcomes in more complex fibrotic scenarios, including radiation-induced lesions and cases with multiple prior interventions [60,61]. Overall, fibrotic and non-lifting benign lesions represent a key indication for EID, particularly when conventional ESD is not feasible. Although current evidence remains limited, the available data are encouraging and support its potential role as an effective organ-preserving option in complex rectal scenarios.

### 5.3. Residual or Recurrent Lesions After Chemoradiotherapy

The conventional approach to treating locally advanced rectal cancers (LARCs), commonly defined as cT3 or cT4 tumors or tumors with nodal involvement, includes neoadjuvant chemoradiotherapy (nCRT) followed by surgical resection and adjuvant CRT. However, increasing interest in nonoperative strategies following nCRT or total neoadjuvant therapy (TNT) has led to the adoption of organ-preserving approaches in selected patients [62]. Specifically, the term “residual rectal neoplasia” indicates the remnant tissue after definitive CRT. In these cases, salvage endoscopic resection is emerging as a viable option when no invasive disease is detected on MRI or endoscopy [62]. Furthermore, emerging evidence suggests that lesions detected after CRT are often biologically less aggressive. In a study by Rupinski et al. [63], lesions that appeared clinically benign after nCRT treatment were confirmed as benign or low-grade neoplasia in the majority of cases, with only a minority harboring invasive cancer. In addition, adenomatous (benign) residuals are less sensitive to nCRT; thus, endoscopic treatment may be a valuable option to achieve optimal outcomes [64]. Among patients managed with a watch-and-wait strategy after complete clinical response, local regrowth occurs in approximately 25% of cases, most commonly as intraluminal lesions, amenable to local endoscopic treatment [29,65,66,67].

These findings support the concept that endoscopic treatment of mostly benign residual and recurrent lesions may be sufficient in selected patients, potentially avoiding radical surgery. Salvage ESD in LARC after nCRT has been shown to be feasible, with 75% R0 resection rates and no adverse events in one series by Leung et al. [68]. However, endoscopic resection in these settings is often technically challenging as CRT induces significant tissue changes, including submucosal fibrosis, vascular alterations, tissue edema, and distortion of the normal rectal wall layers, which may be associated with poor lifting, unclear dissection planes, and an increased risk of incomplete resection or adverse events such as perforation [69,70]. In this setting, EID represents an attractive option, as it directly addresses the key limitation of conventional ESD by bypassing the fibrotic and irradiated submucosa, enabling a more stable and controlled resection, even in the presence of severe post-radiation changes. A recent report demonstrated the feasibility of salvage EID achieving R0 resection in residual rectal adenocarcinoma after CRT, supporting its potential role as an organ-preserving strategy [71]. While these preliminary experiences suggest technical feasibility and potential oncological radicality, further investigations are needed to validate EID in this setting.

## 6. Proposed Role of EID Within the Therapeutic Algorithm of Early Rectal Cancer

Conceptually, EID represents an intermediate approach between local treatment strategies and radical surgery in selected patients with early rectal cancer. However, its precise role in clinical therapeutic algorithms remains to be defined. EID appears particularly suitable for lesions with suspected deep submucosal invasion, muscle-retracting signs or severe fibrosis, and for residual tumors after chemoradiotherapy, in which standard ESD may be insufficient to achieve an adequate vertical margin, while upfront TME could represent overtreatment. Moreover, by providing high-quality en bloc specimens, including the deep invasive front and part of the muscularis propria, EID may represent a valuable option for selected patients considered unsuitable for surgery to obtain local disease control. Nevertheless, EID should not be considered a replacement for oncological surgery, as it cannot guarantee lymph node assessment.

Current evidence does not allow the definition of a validated EID-specific surveillance interval. For patients managed with active surveillance after EID, follow-up may reasonably mirror the strategy used in the Dutch EID cohort, including endoscopic scar assessment, imaging, and CEA monitoring over five years according to national colorectal cancer guidelines [72]. In contrast, when EID or local scar resection is performed after chemoradiotherapy, surveillance may be adapted from organ-preserving rectal cancer protocols, such as those proposed by ESMO, with close multimodal assessment using DRE, endoscopy, and pelvic MRI, especially during the first two years.

**Figure 2:**
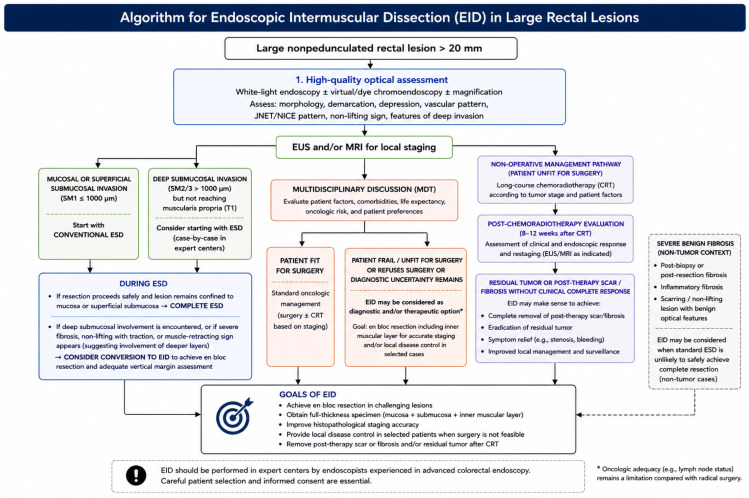
**Proposed algorithm for endoscopic intermuscular dissection (EID) within the management of Large Rectal Lesions**. EID may represent an intermediate organ-preserving approach between conventional endoscopic resection and radical surgery. The figure illustrates how optical diagnosis, EUS/MRI staging, and multidisciplinary assessment can guide patient selection and identify clinical scenarios in which EID may improve local staging accuracy and facilitate rectal preservation while maintaining oncologic appropriateness.

At present, EID should be regarded as an advanced technique to be performed in expert centers within a multidisciplinary setting, integrating high-quality optical diagnosis with EUS and MRI staging to optimize treatment selection and pursue an organ-preserving strategy. The above-mentioned algorithms are summarized in Figure 2.

## Figures and Tables

**Table 1:** Overview of cited studies assessing the diagnostic performance of EUS and MRI for locoregional staging of rectal cancer.

				T Stage			N Stage		
Author	Year	Study Type	Imaging Modality	Sensitivity% (95% CI)	Specificity% (95% CI)	Accuracy% (95%CI)/AUC	Sensitivity% (95% CI)	Specificity% (95% CI)	Accuracy% (95% CI)/AUC
Fernandes MC et al. [29]	2025	Retrospective	*MRI*	-	-	-	56.5% (34.5–76.8)	91.3%(79.2–97.6)	79.7% (68.3 88.4)
Daca-Alvarez M et al. [12]	2026	Multicentre retrospective	*EUS*	-	-	T1: 59%	0% (0.0–48.0)	96.5% (90.8–98.9)	-
Daca-Alvarez M et al. [12]	2026	Multicentre retrospective	*MRI*	-	-	T1: 28.3%	15.8% (4.2–40.5)	91.1% (86.3–94.3)	-
Daca-Alvarez M et al. [12]	2026	Multicentre retrospective	*MRI + EUS*	-	-	32.9%	13% (3.4–34.7)	90.4% (85.7–93.7)	78%
Arndt K et al. [24]	2023	Retrospective	*MRI*	T1: 9% T2: 64%	T1: 79% T2: 38%	35%	N0: 100% N1: 0% N2: 0%	N0: 0% N1: 100% N2: 100%	66%
Rosén R et al. [28]	2022	Retrsopective national cohort	*MRI*	-	-	80.7% (75.2–85.5)	-	-	-
Zhuang Z et al. [23]	2021	Meta-analysis	*MRI*	-	-	-	73% (68–77)	74% (68–80)	-
Chan BPH et al. [30]	2019	Meta-analysis	*EUS*	79% (72–85)	89% (84–93)	-	81% (71–89)	88% (80–94)	-
Chan BPH et al. [30]	2019	Meta-analysis	*MRI*	79% (72–85)	85% (79–90)	-	83% (73–90)	90% (82–95)	-
Puli SR et al. [34]	2009	Meta-analysis	*EUS*	T1: 87.8% (85.3–90.0) T2: 80.5% (77.9–82.9) T3: 96.4% (95.4–97.2) T4: 95.4% (92.4–97.5)	T1: 98.3% (97.8–98.7) T2: 95.6% (94.9–96.3) T3: 90.6% (89.5–91.7) T4: 98.3% (97.8–98.7)	-	-	-	-

This table summarizes the diagnostic performance of EUS and MRI for locoregional staging (T and N) in patients with rectal cancer across included studies. Reported values include sensitivity, specificity, and accuracy with corresponding 95% confidence intervals (CIs), when available. When applicable, stage-specific estimates (e.g., T1–T4 or N categories) are reported within the corresponding cells. A dash (-) indicates that the corresponding data were not reported in the original study. For studies not reporting accuracy directly, area under the curve (AUC) values were reported in the accuracy column. Abbreviations: **CI**: confidence interval; **EUS**, endoscopic ultrasound; **MRI**, magnetic resonance imaging; AUC, area under the curve. “-” indicates data not reported.

## Data Availability

No new data were created or analyzed in this study. Data sharing is not applicable to this article.

## Abbreviations

The following abbreviations are used in this manuscript:

AJCC	American Joint Committee on Cancer
AUC	Area under the curve
CRC	Colorectal cancer
CRT	Chemoradiotherapy
EID	Endoscopic intermuscular dissection
ERUS	Endorectal ultrasound
ESD	Endoscopic submucosal dissection
EUS	Endoscopic ultrasound
kaFTRD	Endoscopic full-thickness resection
LARC	Locally advanced rectal cancer
MRS	Muscle-retracting sign
MRI	Magnetic resonance imaging
NBI	Narrow-band imaging
nCRT	Neoadjuvant chemoradiotherapy
NPV	Negative predictive value
PAEM	Peranal endoscopic myectomy
PCM	Pocket-creation method
RCT	Randomized controlled trial
TEM	Transanal endoscopic microsurgery
TAMIS	Transanal minimally invasive surgery
TME	Total mesorectal excision
TNT	Total neoadjuvant therapy
